# NMN Maintains Intestinal Homeostasis by Regulating the Gut Microbiota

**DOI:** 10.3389/fnut.2021.714604

**Published:** 2021-07-29

**Authors:** Pan Huang, Anqi Jiang, Xuxin Wang, Yan Zhou, Weihong Tang, Caifang Ren, Xin Qian, Zhengrong Zhou, Aihua Gong

**Affiliations:** School of Medicine, Jiangsu University, Zhenjiang, China

**Keywords:** NMN, gut microbiota, colon, bacterial metabolites, intestinal mucosa

## Abstract

The aim of this study was to determine the effects of long-term Nicotinamide mononucleotide (NMN) treatment on modulating gut microbiota diversity and composition, as well as its association with intestinal barrier function. In this study, C57BL/6J mice were fed different concentrations of NMN, and their feces were collected for detection of 16S rDNA and non-targeted metabolites to explore the effects of NMN on intestinal microbiota and metabolites. The results revealed that NMN increased the abundance of butyric acid-producing bacteria (Ruminococcae_UCG-014 and Prevotellaceae_NK3B31_group) and other probiotics (Akkermansia muciniphila), while the abundance of several harmful bacteria (Bilophila and Oscillibacter) were decreased after NMN treatment. Meanwhile, the level of bile acid-related metabolites in feces from the G1 group (0.1 mg/ml) was significantly increased compared to the control group, including cholic acid, taurodeoxycholic acid, taurocholic acid, glycocholic acid, and tauro-β-muricholic acid. In addition, long-term NMN treatment affected the permeability of the intestinal mucosa. The number of goblet cells and mucus thickness increased, as well as expression of tight junction protein. These results demonstrate that NMN reduced intestinal mucosal permeability and exerts a protective effect on the intestinal tract. This study lays the foundation for exploring NMN's utility in clinical research.

## Introduction

As early as 1906, nicotinamide adenine dinucleotide (NAD^+^) was known to increase the fermentation rate of yeast extract, which subsequently became a hot spot in biological research. In recent years, the two intermediates of NAD^+^, NMN and nicotinamide riboside (NR), have received renewed attention (1). NMN is synthesized by niacinamide (a form of water-soluble vitamin B_3_) and 5′-phosphoribosyl-1-pyrophosphate (PRPP). This process is catalyzed by the NAD^+^ biosynthetic rate-limiting enzyme nicotinamide phosphoribosyltransferase (NAMPT) (2, 3). Recent studies have shown that NMN increases concentrations of NAD^+^ in the pancreas, liver and other tissues (4). In addition, long-term (1-year) oral administration of NMN (up to 300 mg/kg) did not cause any obvious deleterious or toxic effects (5). NAD^+^ depletion can lead to a wide range of age-related problems, including neurodegenerative diseases, such as Alzheimer's and Parkinson's, cardiovascular disease and muscle wasting (1, 6–8). In addition to this, NAD^+^ could stimulate intestinal goblet cells to secrete mucus to maintain the integrity of intestinal mucosa, proving that NAD^+^ could protect intestinal homeostasis to a certain extent (9).

The human intestinal mucosa covers an area of up to 200–300 square meters and contains 10 trillion different symbionts, known as the “microbiota”. Microbial communities outnumber somatic and germ cells by more than 10 to one. The genome of the microbiome, known as the microbiome, is 150 times larger than the human genome (10, 11). It not only plays a vital role in the digestive function of the body, but also affects the operation of other systems. In recent years, due to its importance and potential value, the gut microbiota has become a hot topic. The basic functions of the gut microbiota include facilitating the decomposition of food, making it easier to absorb and digest (12), synthesizing essential vitamins (13), removing toxic compounds (14), resisting pathogens (15), maintaining the integrity of the intestinal mucosa (16), and regulating immune function (17). In addition, damage to the intestinal mucosa and imbalance in the gut microbiota can cause invasion of microbial communities into mucosal cells, change their circadian rhythm, and affect lipid absorption and storage, inducing the development of metabolic diseases, such as obesity, type 2 diabetes, and non-alcoholic fatty liver disease (18, 19). Although specific effects and mechanisms of these bacteria on lipid and glucose metabolism have not been fully elucidated, they help maintain energy homeostasis in the body. However, due to individual differences, the impact of the gut microbiota on the human body is not clearly or specifically understood (20).

Bile acid is a metabolite of cholesterol in the liver, and it participates in the process of regulating the absorption and metabolism of cholesterol. Primary bile acids are synthesized in liver cells and enter the intestine with bile to promote the digestion and absorption of lipids. Through the action of bacteria in the lower intestine (the small intestine and large intestine), secondary bile acids are formed. About 95% of intestinal bile acids are reabsorbed by the intestinal wall, including active reabsorption and passive absorption. Reabsorbed bile acids and return to the liver via the portal vein, together with the new synthesis of conjugated bile acid imported into the intestines. This process is called the enterohepatic circulation of bile acids (21). In addition, the antibacterial effect of bile acid can also inhibit the excessive proliferation of bacteria, and has a regulatory effect on the gut microbiota (22). The coordination between the gut microbiota and bile acids plays a key role in maintaining the homeostasis of the intestinal environment.

NMN has great potential for regulating metabolism. However, it remains unclear whether and how NMN affects the gut microbiota, related metabolites and colonic epithelial integrity. The present study aimed to investigate the influence of NMN on the diversity and composition of the gut microbiota and its association with intestinal barrier dysfunction.

## Materials and Methods

### Experimental Animals

All experimental procedures were implemented after approval by the animal ethics committee of Jiangsu University. C57BL/6J mice (female, 12-week-old) were purchased from the Laboratory Animal Center of Jiangsu University (Jiangsu, China) and raised aseptically an environment of 22 ± 3°C and humidity of 40–60% (23). After a week of adaptation, mice were randomly divided into five groups of six individuals for a 15-week period. Mice in the control group drank deionized water, and the other groups were supplemented with different concentrations of NMN in their drinking water, including 0.1 mg/mL (G1 group), 0.2 mg/mL (G2 group), 0.4 mg/mL (G3 group), and 0.6 mg/mL (G4 group).

Mice in all groups were given free access to standard food. All mice were sacrificed after 15 weeks. Blood samples were obtained by orbital blood collection, and serum was collected after separation by centrifugation at 3,000 × *g* for 15 min at 4°C. Then, mice were anesthetized with ether and sacrificed by cervical ligation. The colon and liver tissues were separated, partly stored in a −80°C freezer, and partly fixed in 4% paraformaldehyde. The feces in the intestines were removed and stored in liquid nitrogen and then frozen in at −80°C for subsequent 16S rDNA sequencing and untargeted metabolome assay.

### *In vivo* Intestinal Permeability (IP)

To determine the intestinal mucosal barrier permeability, mice were given FITC-dextran 4 kDa (FD4: 500 mg/kg BW; Sigma) orally and anesthetized two hours later for *in vivo* imaging. After 4 hours, blood samples were collected from the orbit and serum was separated. The concentration of FD4 in serum was measured by automatic Infinite M200 microplate reader (Tecan, Austria) (Ex 485 nm; Em 525 nm). Paraffin sections of mouse colon samples were dewaxed and dehydrated, and then the nuclei were stained with DAPI and observed under a microscope.

### Morphological Analysis and Immunohistochemistry

The tissues fixed with 4% paraformaldehyde were washed with phosphate buffer saline (PBS) and dehydrated with 70, 80, 90% ethanol and anhydrous ethanol. After transparent treatment with xylene, the tissues were embedded in paraffin and cut into sections 5 μm thick. The slices were then stained with hematoxylin and eosin (H&E) (SolarBio, G1120) and Alcian Blue (Leagene, DG0041).

The following antibodies were used for immunohistochemistry: anti-claudin-1 (rabbit, polyclonal, 1:200, 13255S; CST, Massachusetts, America), anti-ZO-1 (rabbit, polyclonal, 1/100, 61–7300, Invitrogen), anti-LC3 (rabbit, polyclonal, 1/400, 4599S; CST, Massachusetts, America), and secondary goat anti-rabbit Alexa Fluor 488 antibody (1/2000, Invitrogen). Paraffin sections were incubated overnight with primary antibody at 4°C and then incubated with corresponding secondary antibody at 37°C for 20 min. According to the instructions provided by the manufacturer, the nuclei were stained with hematoxylin after incubated with the DAB. All tissue sections were examined using light microscopy.

### DNA Extraction and PCR Amplification

DNA was extracted from samples using the E.Z.N.A.® Fecal DNA Kit (D4015, Omega, Inc., USA) according to the manufacturer's instructions and stored in a −80°C Refrigerator (24). Samples were sent to LC-Bio (Hang Zhou, China) for PCR amplification. The V4 region of the 16S rDNA subunit of prokaryotes (bacteria and archaea) was amplified by the modified primers 515F (5 ′-GTGYCAGCMGCCGCGGTAA-3′) and 806R (5 ′-GGACTACHVGGTWTCTAAT-3′) and then sorted the libraries on the NovaSeq platform with PE250.

### Intestinal Microbiota Analysis

Samples were sequenced on Illumina NovaSeq platform according to manufacturer's recommendations and provided by LC-Bio. Alpha diversity was applied to the analysis of the complexity of the species diversity, through the five indicators, including Chao1 and Observed species, Goods coverage, Shannon, Simpson. All these indicators were used QIIME2 calculated. Beta diversity is calculated by QIIME2 and plotted by the R package. The sequence was calibrated using Blast, and each representative sequence was annotated by SILVA database. The other diagrams were implemented using the R package (V3.5.2).

### Fecal Sample Preparation for Metabolomics Analysis

After thawing on ice, metabolites were extracted with 50% methanol buffer. The extract was stored overnight in a refrigerator at −20°C. After centrifugation at 4000 g for 20 min, the supernatant was taken and stored at −80°C (25).

### LC-MS/MS Data Acquisition

All chromatographic separations were performed using an ExionLC system (SCIEX, Framingham, MA, USA). An ACQUITY UPLC T3 column (100 mm^*^ 2.1 mm, 1.8 μm, Waters, UK) was used for reverse phase separation. The metabolites of column elution were detected by high resolution tandem mass spectrometry (TripleTOF5600 plus) (SCIEX, Framingham, MA, USA). See the references for specific parameter Settings (26).

### Statistical Analysis

Data are expressed as means ± SEM. An unpaired two-tailed Student *t*-test was used to assess differences between the two groups. Data sets involving more than two sets were evaluated using Kruskal-Wallis test. Correlations were analyzed using Spearman Correlation Coefficient. Data were analyzed using GraphPad Prism version 8.0 (GraphPad Software). At *P* < 0.05, a result was considered statistically significant.

## Results

### The Effect of Long-Term NMN Treatment on the Body Weight of Mice

**Figure 1:**
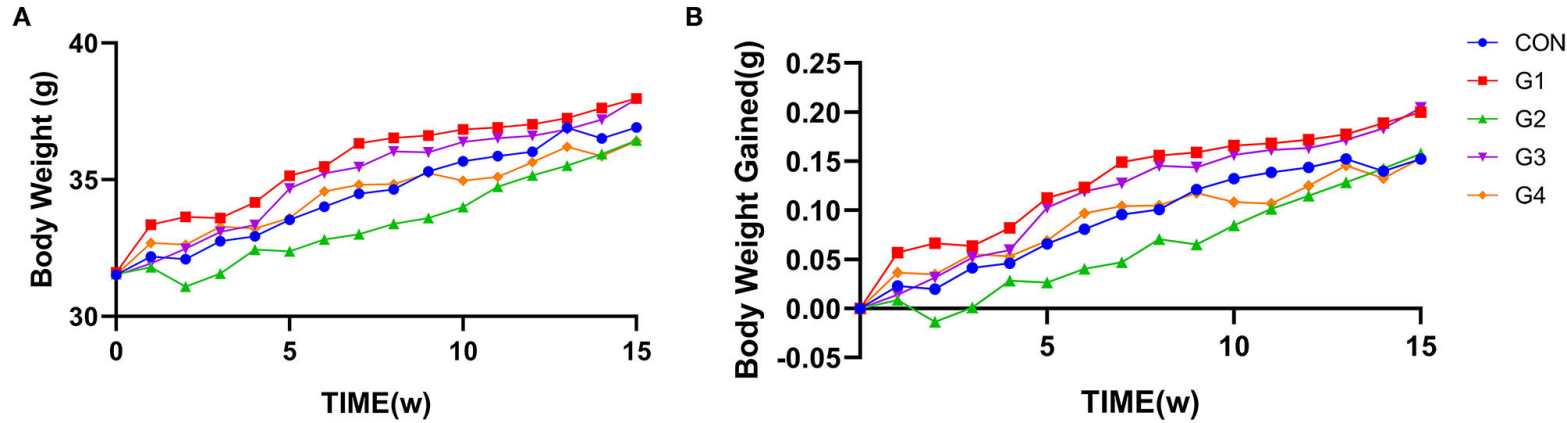
The effect of long-term NMN treatment on body weight in mice. Long-term supplementation of NMN had no significant effect on body weight **(A)** or the body weight change rate **(B)** between the two groups of mice.

Mice were supplemented with NMN for 15 consecutive weeks. Body weight was measured every 7 days, and the weight gain rate was calculated. The results showed that there was no significant difference in body weight or weight gain rate between the two groups of mice (Figure 1).

### The Effects of Long-Term NMN Treatment on the Diversity of the Gut Microbiota

**Figure 2:**
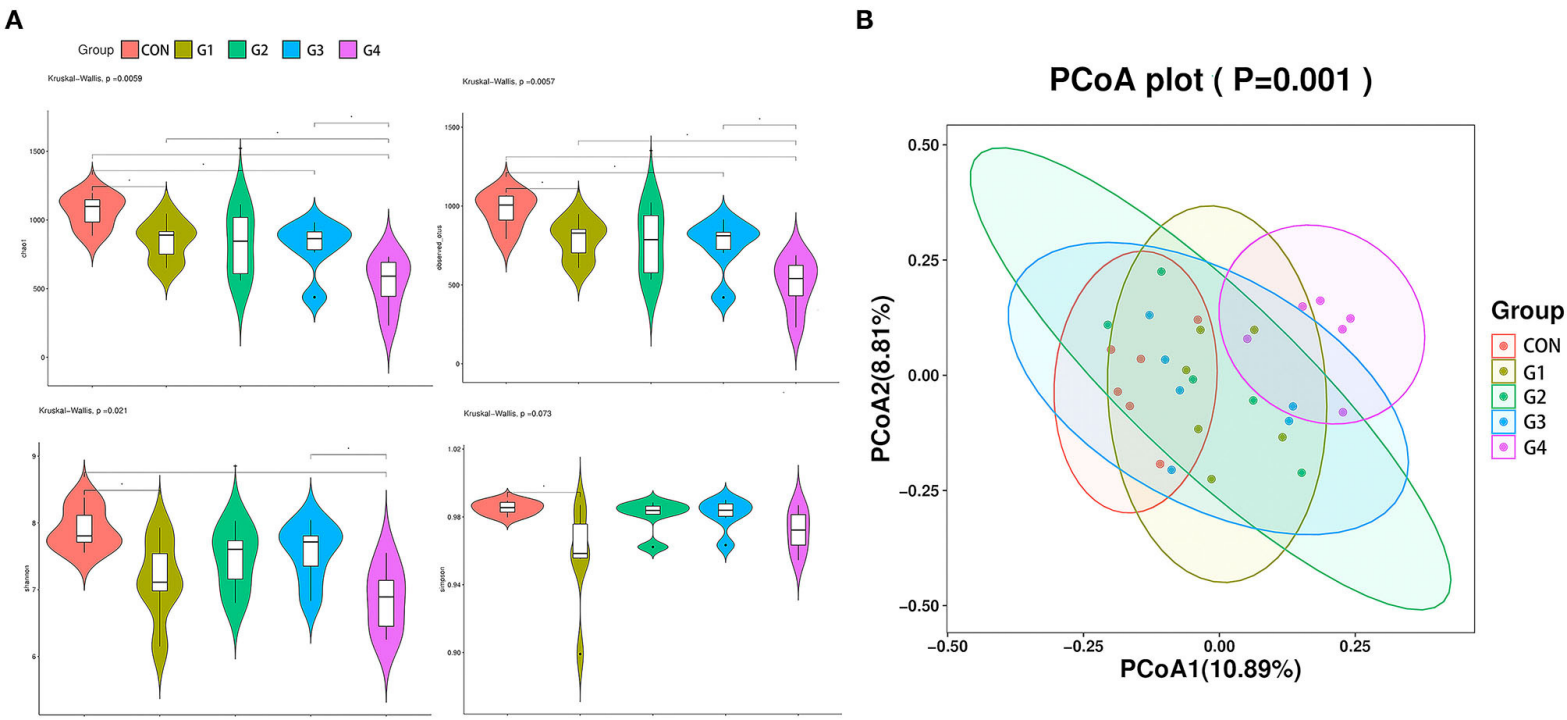
The effects of long-term NMN treatment on the diversity of intestinal flora. α diversity analysis of the feces from mice was performed, and the four indexes were Chao1 **(A)**, Observed **(B)**, Shannon **(C)**, and Simpson **(D)** (*P < 0.05, **0.001 < *P* < 0.01). β diversity analysis of gut microbiota and PCOA analysis **(E)** of fecal flora composition in each group (*P* = 0.001).

To analyze changes in the diversity of the gut microbiota in each group, we examined several indicators of alpha diversity. The chao1 index and observed species (Figures 2A,B) index primarily reflect the number of OTU species, while the Shannon and Simpson (Figures 2C,D) indexes are relative to the average and homogeneity. The results showed that after administration of NMN, the chao 1 index and other observed species indexes were decreased and was negatively correlated with the concentration of NMN. Surprisingly, NMN reduced the diversity of intestinal species. Principal coordinate analysis (PCoA) was performed to evaluate the comparability of microbial communities among the five groups. PCoA results showed significant differences between the G4 group and the CON group, indicating a significant difference in microbial composition (Figure 2E). These results indicated that 0.6 mg/mL of NMN had a significant effect on the microbial composition of mouse feces. Figure 2B shows that the primary principal component and secondary principal component account for 10.89 and 8.81% of the overall analysis results, respectively.

### Effects of Long-Term NMN Treatment on Species Abundance in the Gut Microbiota

**Figure 3:**
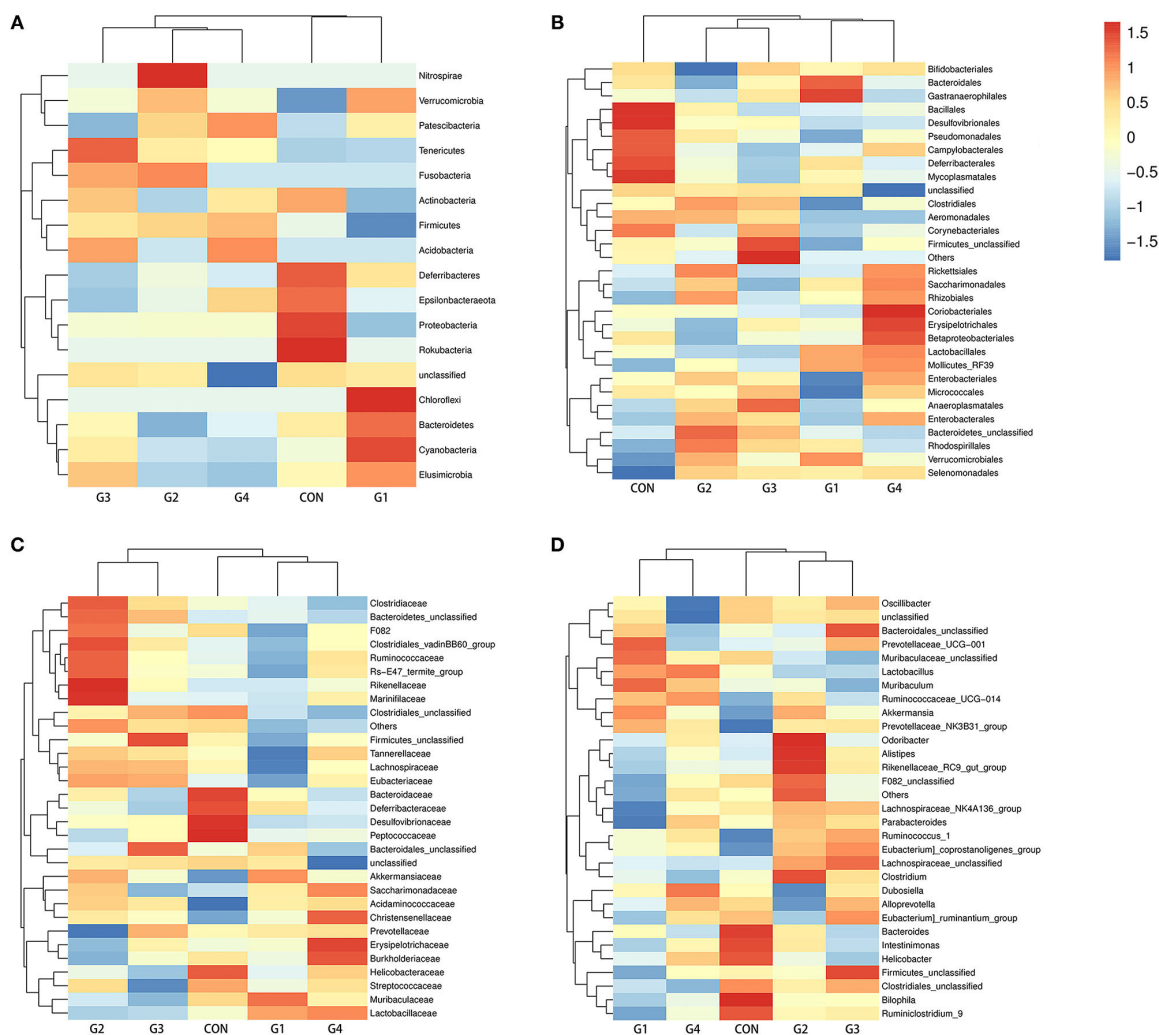
The effects of long-term NMN treatment on the structure of intestinal microflora. Abundance analysis of gut microbiota population, shown as heat maps of species abundance at phylum **(A)**, order **(B)**, family **(C)**, and genus **(D)** levels.

**Figure 4:**
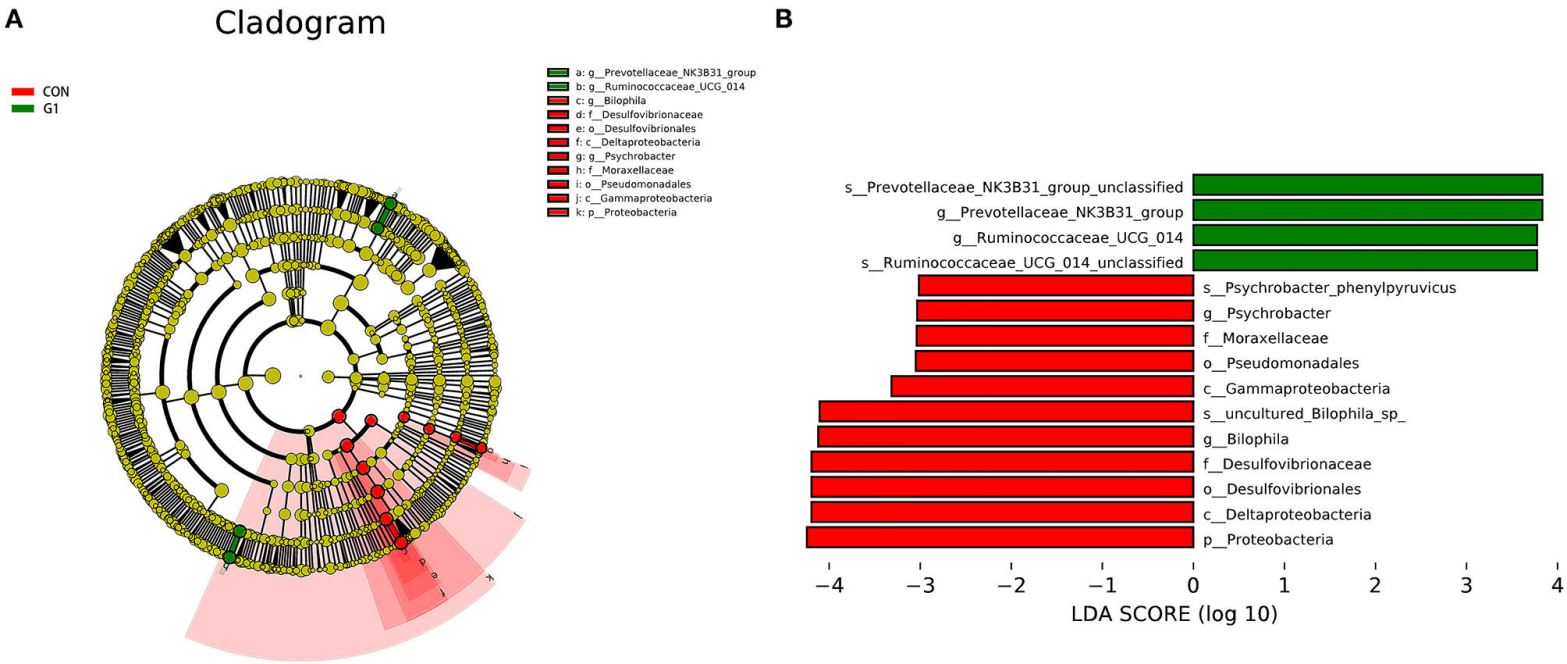
Cladogram indicating the phylogenetic distribution of microbiota correlated with each group **(A)**. The differences in abundance between each group **(B)**.

As the concentration of NMN increased, the diversity of the intestinal flora gradually decreased, so we compared the G1 group (supplemented with 0.1 mg/ mL NMN) to the control group. To compare differences in fecal microflora, Welch's *t*-test was performed for different classification levels. At the phylum level, the abundance of Bacteroidetes, Verrucomicrobia, Patescibacteria, Cyanobacteria and Elusimicrobia was higher in the G1 group than in the control group, while the abundance of Firmicutes, Proteobacteria, Epsilonbacteraeota, Deferribacteres, Actinobacteria and some unclassified bacteria was lower in the G1 group than that in the control group (Figure 3A). Among them, only Proteobacteria (*P* < 0.05) was significantly different (Figure 4).

At the order level, the abundance of Bacteroidales, Lactobacillales, Verrucomicrobiales, Erysipelotrichales, Saccharimonadales, Bacteroidetes_unclassified, Selenomonadales, Gastranaerophilales, Rhodospirillales, Mollicutes_RF39 and Rhizobiales was higher in the G1 group than in the control group, while the abundance of Clostridiales, Desulfovibrionales, Firmicutes_unclassified, Campylobacterales, Betaproteobacteriales, Deferribacterales, Anaeroplasmatales, Coriobacteriales, Corynebacteriales, Pseudomonadales, Enterobacteriales, Aeromonadales, Bifidobacteriales, Bacillales, Rickettsiales, Micrococcales, and Mycoplasmatales was lower in the G1 group than in the control group (Figure 3B). In addition, Aeromonadales (*P* < 0.05), Flavobacteriales (*P* < 0.05), Desulfovibrionales (*P* < 0.05), Mollicutes_RF39 (*P* < 0.05), Pseudomonadales (*P* < 0.05) and Bifidobacteriales (*P* < 0.05) levels were significantly different between the two groups (Figure 4).

At the family level, the abundance of Mollicutes_RF39_unclassified, Muribaculaceae, Prevotellaceae, Lactobacillaceae, Bacteroidales_unclassified, Akkermansiaceae, Erysipelotrichaceae, Saccharimonadaceae, Bacteroidetes_unclassified, Acidaminococcaceae and Christensenellaceae was higher in the G1 group than in the control group, and the abundance of Aerococcaceae, Moraxellaceae, Aeromonadaceae, Atopobiaceae, Bifidobacteriaceae, Lachnospiraceae, Ruminococcaceae, Bacteroidaceae, Rikenellaceae, Clostridiales_unclassified, Desulfovibrionaceae, Firmicutes_unclassified, Clostridiaceae, Tannerellaceae, Helicobacteraceae, Marinifilaceae, Burkholderiaceae, Streptococcaceae, Deferribacteraceae, Peptococcaceae, and Eubacteriaceae was lower in the G1 group than in the control group (Figure 3C). Among them, Mollicutes_RF39_unclassified (*P* < 0.05), Aerococcaceae (*P* < 0.01), Moraxellaceae (*P* < 0.05), Aeromonadaceae (*P* < 0.05), Desulfovibrionaceae (*P* < 0.05), Atopobiaceae (*P* < 0.05), Bifidobacteriaceae (*P* < 0.05) were significantly different (Figure 4).

At the genus level, the abundance of Muribaculaceae_unclassified, Lactobacillus, Bacteroidales _unclassified, Akkermansia, Lachnospiraceae_unclassified, Muribaculum, Ruminococcaceae_UCG-014, Prevotellaceae_UCG-001, Ruminococcus_1, Dubosiella, and Prevotellaceae_NK3B31_group was higher in the G1 group than in control group, and the abundance of Lachnospiraceae_NK4A136_group, Bacteroides, Alloprevotella, Clostridiales_unclassified, Firmicutes_unclassified, Clostridium, Alistipes, Parabacteroides, Bilophila, F082_unclassified, Rikenellaceae_RC9_gut_group, Ruminiclostridium_9, Intestinimonas, Oscillibacter, Helicobacter, and Odoribacter was lower in the G1 group than in the control group (Figure 3D). A total of 17 genera were significantly different between the two groups, including Ruminococcaceae_UCG-014 (*P* < 0.01), Facklamia (*P* < 0.01), Paenalcaligenes (*P* < 0.01), Coriobacteriaceae_UCG-002 (*P* < 0.01), Desulfovibrionaceae (*P* < 0.01), Bilophila (*P* < 0.01), Peptococcus (*P* < 0.01), Corynebacterium_1 (*P* < 0.05), Psychrobacter (*P* < 0.05), Oceanisphaera (*P* < 0.05), Prevotellaceae_NK3B31_group (*P* < 0.05), Jeotgalicoccus (*P* < 0.05), Ruminococcus (*P* < 0.05), Aerococcus (*P* < 0.05), Candidatus_Stoquefichus (*P* < 0.05), Bifidobacterium (*P* < 0.05). The above results indicate that NMN can regulate the diversity of gut microbiota and improve its structure (Figure 4). The data presented in the study are deposited in the SRA repository, accession number PRJNA739491.

### The Effects of Long-Term NMN Treatment on the Concentration of Common Metabolites in Feces

**Figure 5:**
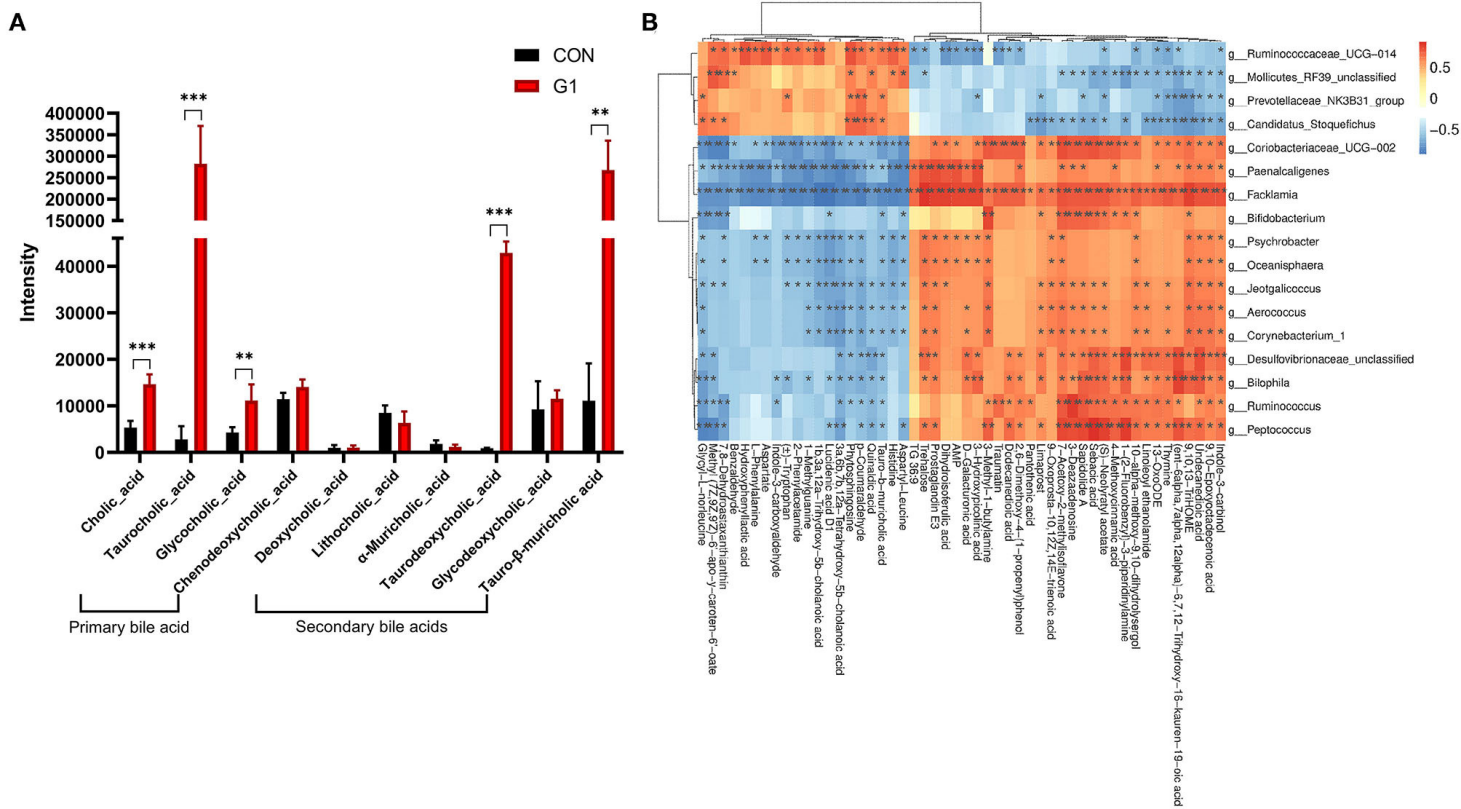
The effects of long-term NMN treatment on the concentration of bile acid-related metabolites. The concentration of bile acid-related metabolites was significantly increased **(A)** *0.01 < *P* < 0.05, **0.01 < *P* < 0.001, and ***P < 0.001. Correlation analysis of differential bacteria and metabolites **(B)**.

After observing the effects of NMN on gut microbiota, we performed an untargeted metabolome assay. As species diversity in the G1 group was relatively rich, and the abundance of beneficial bacteria was the highest, the G1 and control groups were selected for further analysis. Compared to the control group, the content of bile acid-related metabolites was significantly altered in the G1 group, with the content of primary bile acids and secondary bile acids being significantly increased in the G1 group (Figure 5A), including cholic acid (CA) (*P* < 0.0001), taurodeoxycholic acid (TDCA) (*P* < 0.001), taurocholic acid (TCA) (*P* < 0.01), glycocholic acid (GCA) (*P* < 0.01), and tauro-β-muricholic acid (TMCA) (*P* < 0.01).

Based on the above sequencing and metabolite results, we conducted a correlation analysis between differential bacteria and metabolites (Figure 5B). The results showed that TCA and GCA were positively correlated with Ruminococcaceae_UCG-014, Candidatus_Stoquefichus, Mollicutes_RF39_unclassified and Prevotellaceae_NK3B31_group and negatively correlated with Facklamia, Paenalcaligenes, Coriobacteriaceae_UCG-002, Desulfovibrionaceae_unclassified, Bilophila, Peptococcus, Ruminococcus, and Bifidobacterium. In addition, TMCA was positively correlated with Ruminococcaceae_UCG-014, Candidatus_Stoquefichus and Prevotellaceae_NK3B31_group and negatively correlated with Facklamia, Paenalcaligenes, Coriobacteriaceae_UCG-002, Desulfovibrionaceae_unclassified, Bilophila, Peptococcus, Corynebacterium_1, Psychrobacter, Oceanisphaera, Aerococcus, Jeotgalicoccus, Ruminococcus, and Bifidobacterium.

**Figure 6:**
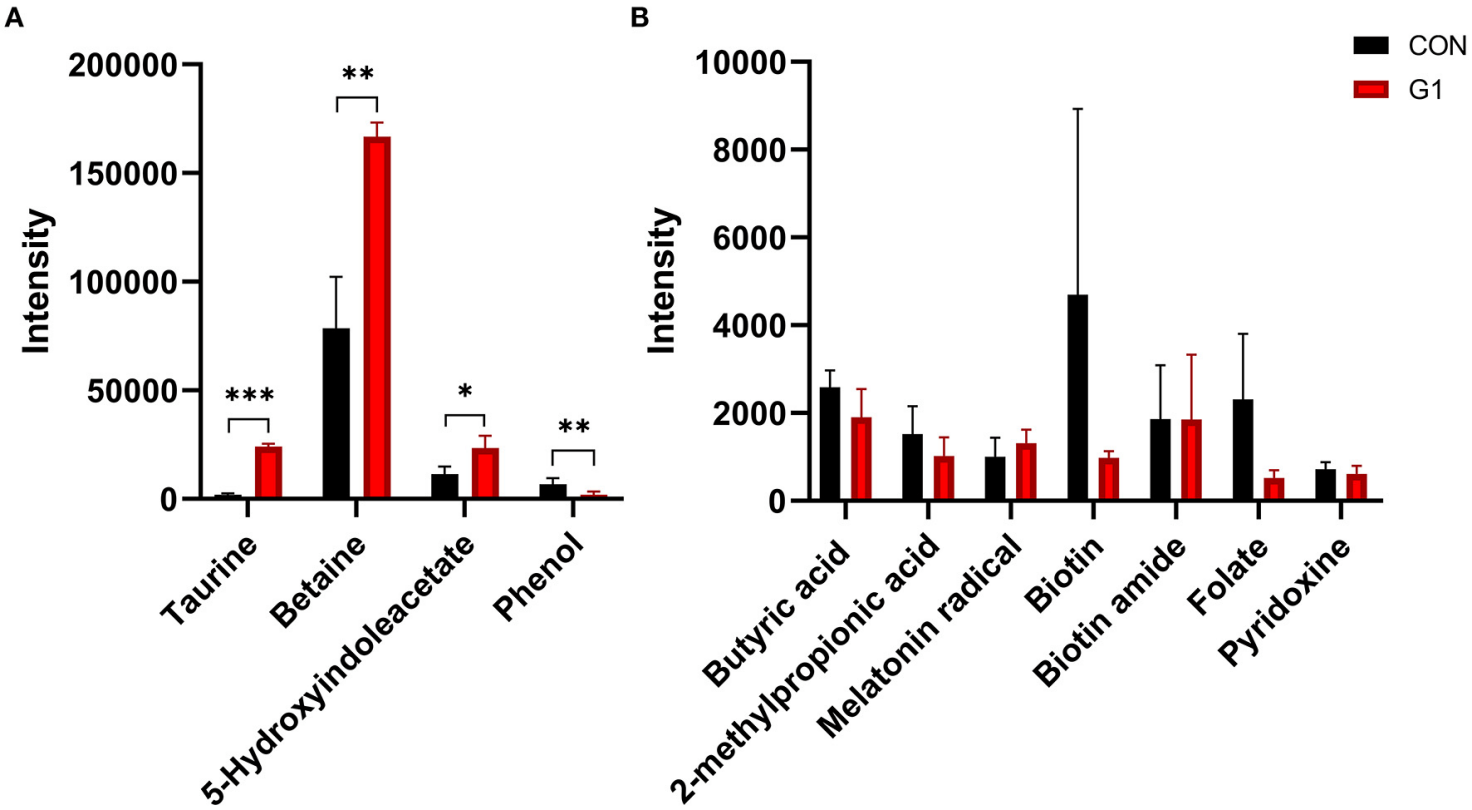
The effects of long-term NMN treatment on other common metabolites. The concentration of some common metabolites was significantly increased **(A)**, others were not affected **(B)** *0.01 < *P* < 0.05, **0.01 < *P* < 0.001, and ***P < 0.001.

Meanwhile, there were other differential metabolite levels between the two groups, such as taurine (*P* < 0.0001), betaine (*P* < 0.001), phenol (*P* < 0.01), and 5-hydroxyindoleacetate (*P* < 0.05) (Figure 6A). These include some common metabolites, such as butyric acid, which was not significantly different between the two groups (Figure 6B).

Taken together, NMN was observed to increase the level of bile acid-related metabolites and beneficial metabolites, such as betaine, and decrease levels of phenol by affecting the composition of the gut microbiota.

### The Effect of Long-Term NMN Treatment on Serum NAD^+^ Concentrations

**Figure 7:**
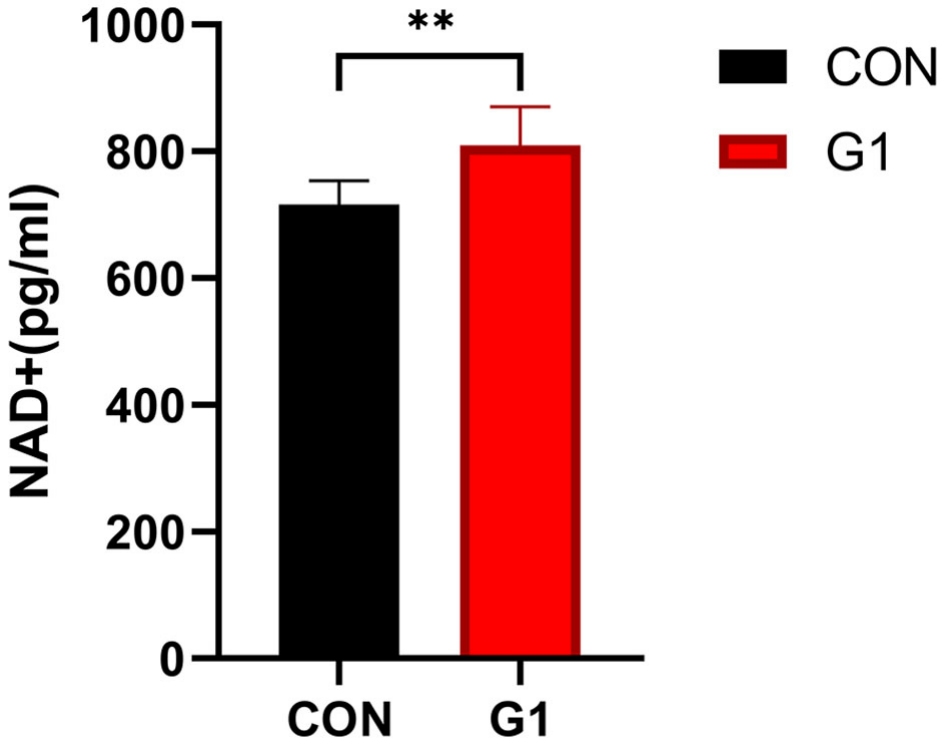
Serum NAD+ content was significantly increased after long-term NMN treatment (*P* < 0.01).

The serum NAD+ concentration of mice in the G1 group was significantly higher than in the control group (*P* < 0.05) (Figure 7), indicating that NMN significantly increased the concentration of NAD+.

### The Effects of Long-Term NMN Treatment on the Morphology of The Intestinal Mucosa

**Figure 8:**
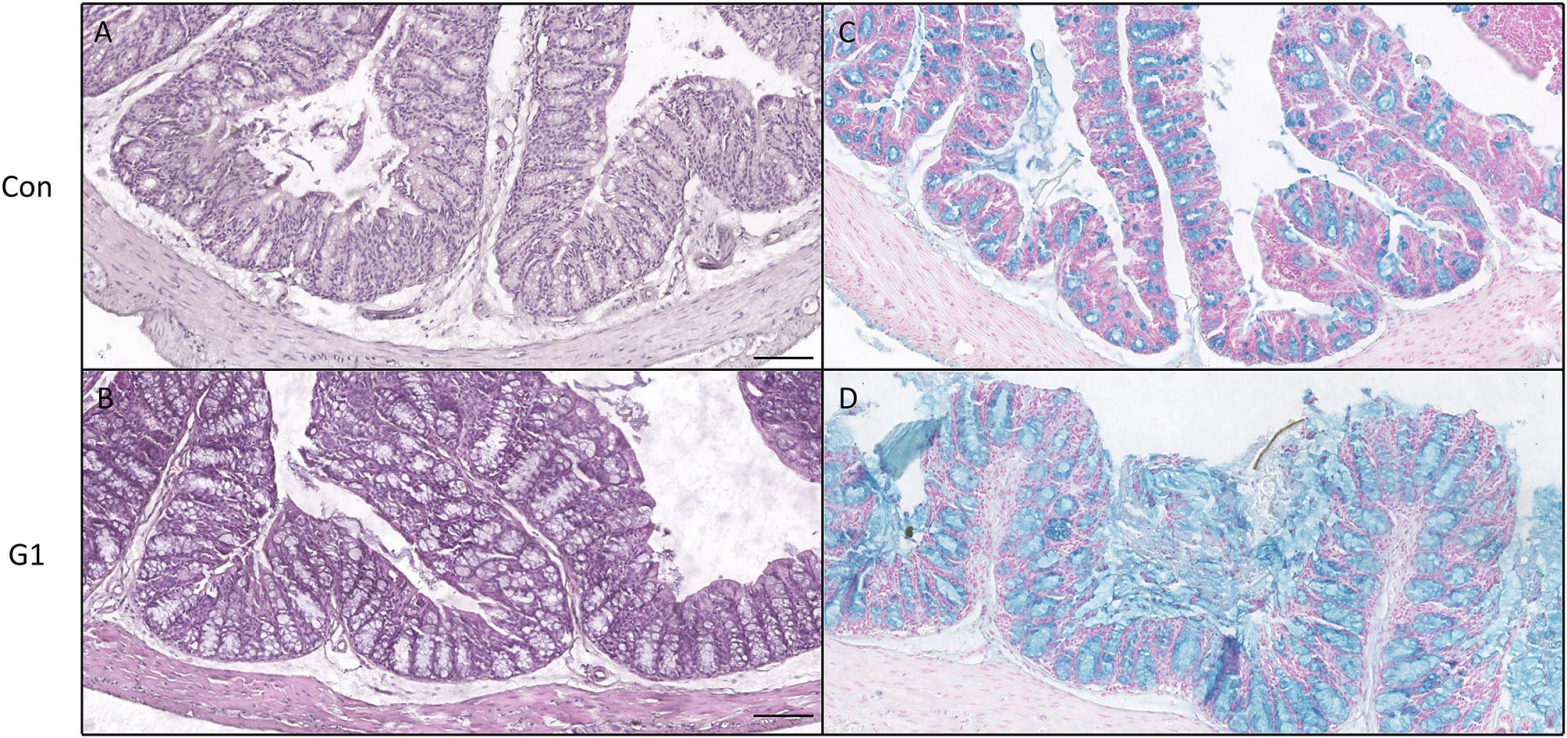
The effects of long-term NMN treatment on colon morphology. H&E staining **(A,B)** and Alcian Blue staining for colonic mucus and goblet cells **(C,D)**, bar: 100 μm.

As presented in Figures 8A,B, no significant difference in morphology was observed in H&E staining. However, further observation by Alcian Blue staining revealed that the number of goblet cells and the thickness of mucus in the G1 group were significantly increased compared to the control group (Figures 8C,D). Thus, NMN increased the number of goblet cells and promoted mucus secretion.

### The Effects of Long-Term NMN Treatment on the Intestinal Mucosal Barrier

**Figure 9:**
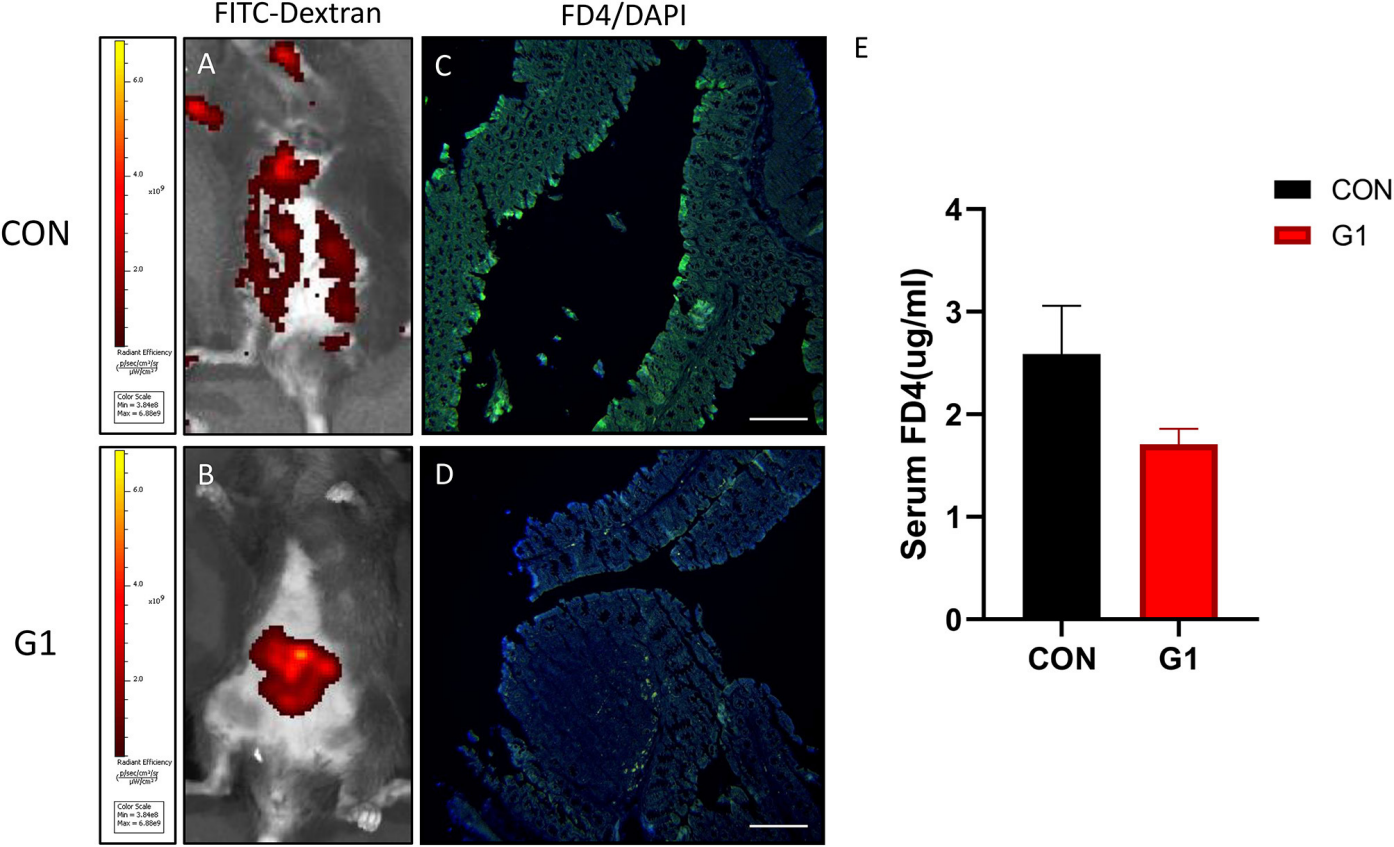
The effect of long-term NMN treatment on intestinal mucosal permeability in mice. Leakage of FD4 was observed by *in vivo* imaging **(A,B)**. Colonic tissue section observing the embedment of FD4 into colon epithelial tissue **(C,D)**; scale bar: 100 μm. Levels of FD4 in the serum **(E)**.

Compared to the control group, the intestinal mucosal permeability in the G1 group was decreased. *In vivo* imaging system of small animals revealed that the leakage area of 4 kDa FITC-labeled dextran (FD4) in the G1 group was significantly less than in the control group (Figures 9A,B). As shown in Figures 9C,D, FD4 colonic immunofluorescence revealed the FD4 infiltration of colonic epithelial cells in the G1 group was less than in the control group. In addition, the serum fluorescence intensity test revealed that the concentration of FD4 in the G1 group was higher than in the control group (*P* > 0.05) (Figure 9E). Taken together, these findings indicate that NMN reduces intestinal mucosal permeability and maintains mucosal barrier integrity.

**Figure 10:**
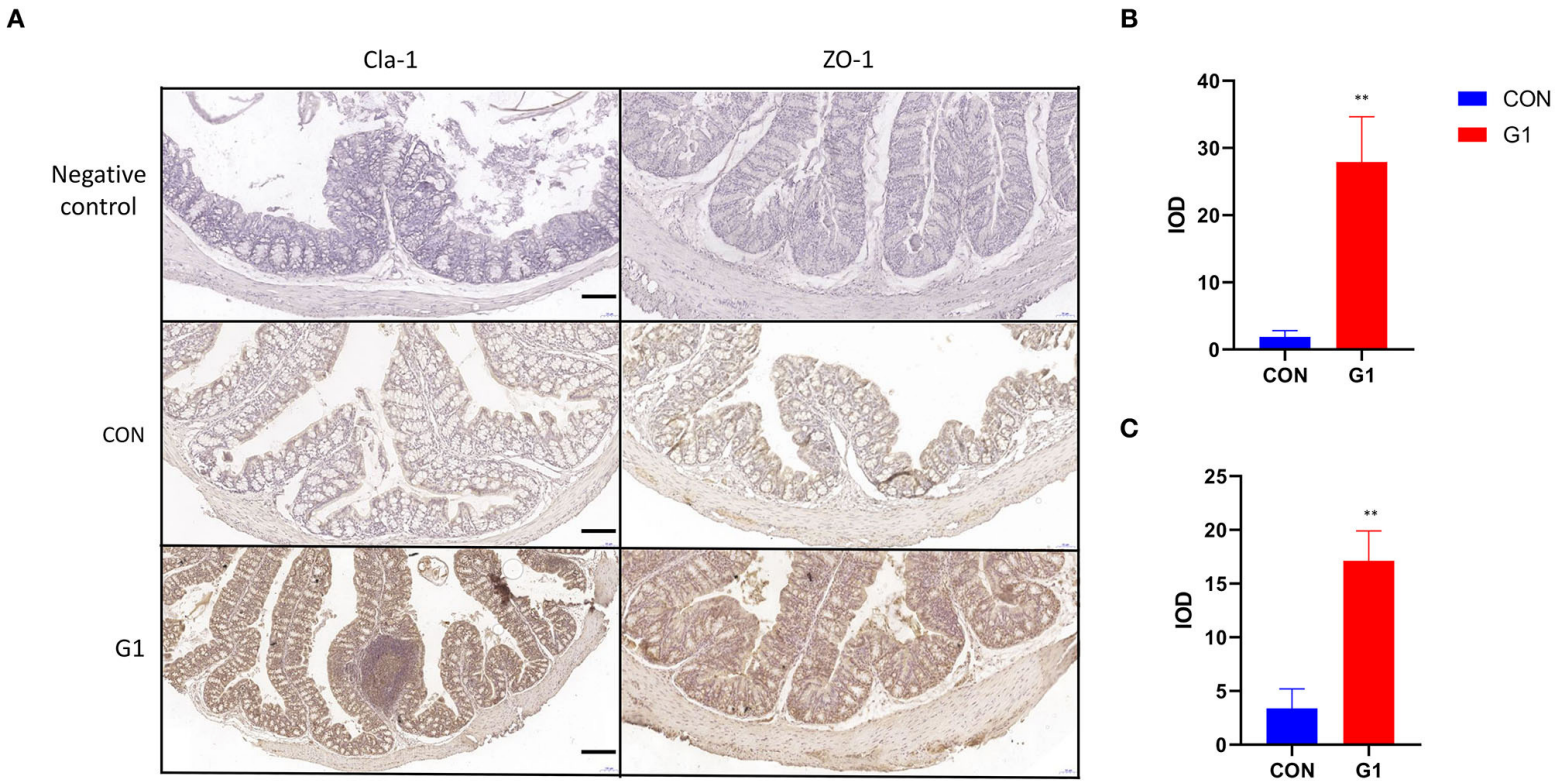
The effect of long-term NMN treatment on the expression and localization of colonic tight junction proteins in mice. The expression of tight junction proteins Claudin-1 and ZO-1 in the colonic epithelium of mice (scale bar: 100 μm) **(A)** after treatment with NMN. Immunohistochemical results of Claudin-1 and ZO-1 were analyzed by Image-Pro Plus **(B,C)** *0.01 < *P* < 0.05, and **0.001 < *P* < 0.01.

To clarify the mechanism of action, we detected the expression and localization of Claudin-1 and ZO-1 in the mucosal epithelium. Expression levels of these two proteins in the G1 group were higher than in the control group, with expression primarily localized in the cytoplasm of epithelial cells (Figure 10A). Next, these immunohistochemical results were analyzed using Image-Pro Plus. The results demonstrated that NMN promotes expression of both Claudin-1 and ZO-1, improving the integrity of the mucosal barrier, but its upstream mechanism remains to be studied (*P* < 0.01) (Figures 10B,C).

### The Effects of Long-Term NMN Treatment on LC3

**Figure 11:**
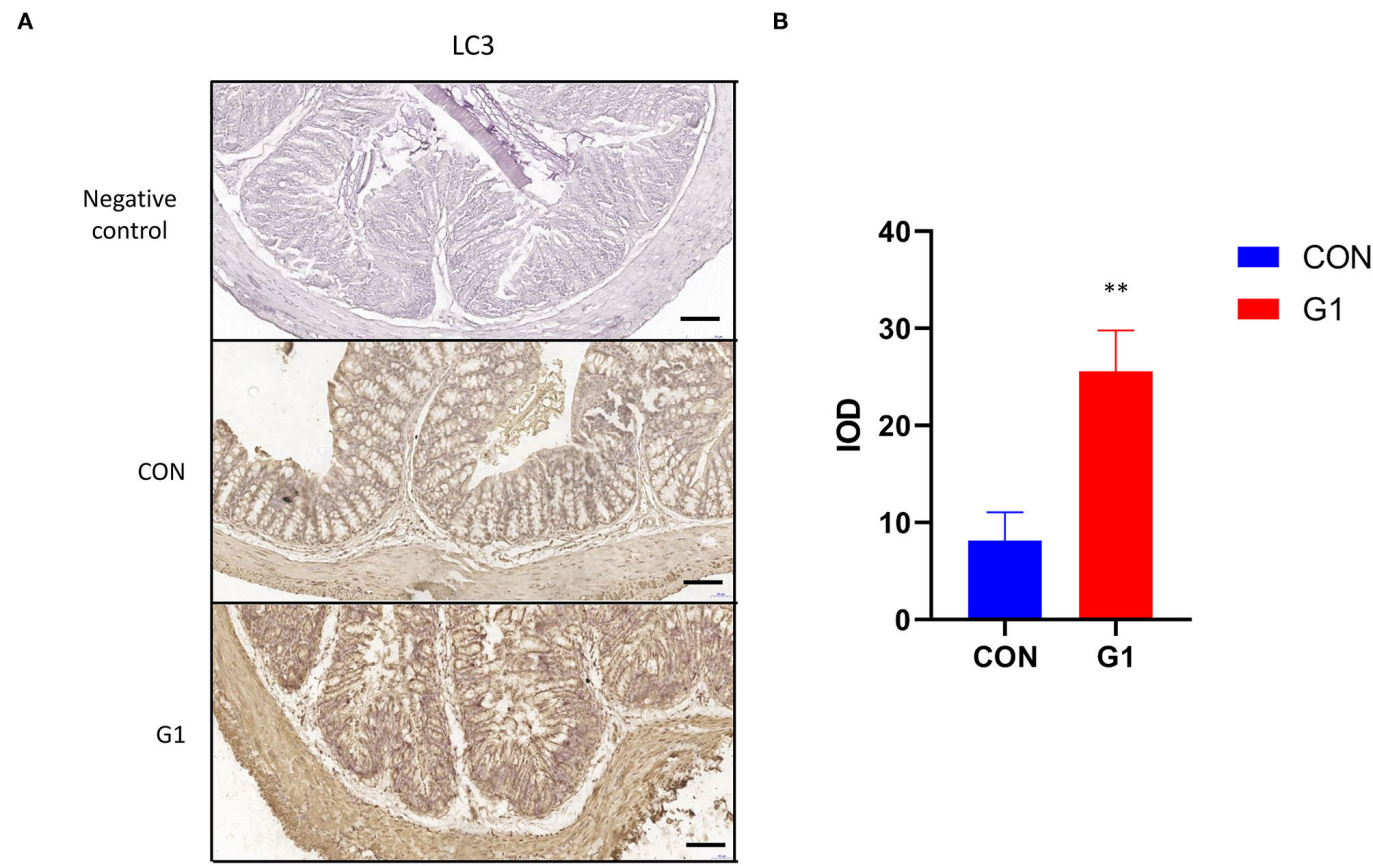
The effects of long-term NMN treatment on the expression and localization of colonic autophagy protein in mice. Expression of the autophagy protein LC3 in colonic epithelium of mice (scale bar: 100 μm) **(A)** after treatment with NMN. The immunohistochemical results of LC3 were analyzed using Image-Pro Plus **(B)** 0.001 < *P* < 0.01.

Expression levels of light chain 3 (LC3) in the G1 group were significantly higher than in the control group, and it was expressed in both the cytoplasm and nucleus (Figure 11A). Image-Pro Plus analysis revealed the same results (*P* < 0.01) (Figure 11B). These results indicate that NMN promotes autophagy.

## Discussion

The sequencing results revealed that NMN increases the abundance of butyrate-producing bacteria, such as Ruminococcaceae_UCG-014 (27) and Prevotellaceae_NK3B31_group. Compared to the control group, the abundance of these two bacteria in the G1 group was significantly increased. Ruminococcaceae is a beneficial microorganism colonized in cecum and colon, which can degrade various polysaccharides and fibers to produce short-chain fatty acids (SCFAs) (28, 29). Butyrate is a short chain fatty acid that produces ketones and carbon dioxide. It is a major energy source for colon cells, and its absence can lead to impaired intestinal barrier function (30). However, the content of butyric acid did not increase. Recent studies have shown that the abundance of Ruminococcaceae in actively drinking alcohol-dependent subjects is decreased while intestinal permeability is increased (31). Moreover, Ruminococcaceae also has anti-inflammatory properties. Zhao et al. found that the abundance of Ruminococcaceae in the feces of obese mice fed a high-fat diet was significantly reduced (32). Taken together, these data indicate that Ruminococcaceae protects intestinal health in many ways.

Except for butyric acid producing bacteria, Akkermansia was increased in the G1 group compared to the control group, but there was no significant difference. Akkermansia muciniphila (*A. muciniphila*), an intestinal symbiotic bacterium that colonizes the mucous layer, is considered a promising probiotic candidate (33). Studies have shown that Akkermansia can degrade mucins (34, 35). In fact, Akkermansia not only had the ability to degrade mucin, but also promoted mucin synthesis, meaning that it could promote mucin renewal (36, 37). In addition, some studies have reported a decrease in the abundance of *A. muciniphila* in various diseases, including diseases of the digestive system, such as IBD (38–41).

Intestinal mucus is composed of highly glycosylated proteins secreted by mucous epithelial cells in the form of large aggregates, composed of a bacteria-free inner layer and a thicker outer layer with commensal bacteria (42). Mucins secreted by goblet cells form a barrier that prevents external bacteria from directly contacting the epithelial layer (42). Its main ingredient, mucins, are asource of nutrients for gut bacteria, because it is made up of amino acids and oligosaccharides. Alcian Blue staining revealed an increase in the number of intestinal goblet cells and the thickness of mucus. Combined with the above results, NMN may protect the integrity of the intestinal mucosa by increasing the abundance of Akkermansia.

In addition to increasing the abundance of certain beneficial bacteria, NMN also reduced the abundance of certain harmful bacteria, such as Bilophila. Compared to the control group, the abundance of Bilophila in the G1 group was significantly decreased. Bilophila was originally isolated from the appendix tissue of patients with gangrene and perforating appendicitis (43) and was subsequently isolated from clinical infection specimens, such as sepsis and cholecystitis (44). This suggests that this bacterium may be related to the occurrence and development of disease. In 2012, Devkota et al. first demonstrated that Bilophila is a cause of IBD (45). NMN is supposed to play a role in improving enteritis. In addition to Bilophila, the abundance of Oscillibacter also exhibited a decreasing trend. Oscillibacter has been reported to be associated with trimethylamino oxide (TMAO), which is a risk factor for cardiovascular and cerebrovascular disease (46). This is also strong evidence that NMN can alleviate cardiovascular disease. Desulfovibrionaceae are Gram-negative bacteria that produce endotoxin (47), primarily including lipopolysaccharides (LPS). LPS is very likely to induce inflammation (48). The abundance of Desulfovibrionaceae in the intestines of mice treated with NMN was significantly reduced, indicating that NMN exerts an inhibitory effect on inflammation.

The concentration of bile acid-related metabolites, including CA, TDCA, TCA, GCA, and TMCA, was significantly increased in the G1 group. Metabolites related to bile acid are closely related to the gut microbiota, and bacteria with 7 α-dehydroxylation in the gut microbiota can convert primary bile acids into secondary bile acids (49). The product of CA, deoxycholic acid (DCA) and its bacterial dehydrogenation have a strong inhibitory effect on the growth of bacterial, and the effect of DCA is 10 times that of CA (22). Thus, gut microbiota faced strong survival selection pressure from bile acids. This may explain why the diversity of gut microbiota decreased after long-term NMN treatment.

Erin et al. demonstrated that adding TDCA to the diet can maintain intestinal mucosal integrity by reducing apoptosis, stimulating cell proliferation and increasing villi length (50). In addition, some studies had found that TDCA could promote the renewal of intestinal mucosa by mediating the up-regulation of c-Myc expression by FXR (51). In other words, NMN maintains the integrity of the intestinal mucosal barrier partly by increasing the concentration of TDCA.

Why was the level of bile acids in the feces significantly higher in response to NMN? First, taurine can promote the proliferation of Bilophila (52). The primary source of taurine in the body is bezoar-bound bile acids. Concentrations of TCA and TDCA in the G1 group were significantly increased, while the abundance of Bilophila was decreased, which is inconsistent with the results of previous studies. Thus, we speculate that the increase in fecal bile acid concentration is not caused by the increase in bile acid synthesis, but by the decrease in ileal reabsorption. Bile acids can be reabsorbed in the small intestine in a variety of ways. The distal ileum is the main site for bile acid reabsorption because the apical sodium-dependent bile acid transporter (ASBT) is mainly expressed here (53). Out et al. found that ASBT-dependent ileal bile acid reabsorption was inhibited in intestinal Gata4 specific knockout mice, which is related to changes in gut microbiota in an ASBT-dependent pathway through Gata4 (54). However, due to the lack of relevant experimental data, the above conjecture needs further research to confirm.

In addition to bile acid-related metabolites, the concentration of other metabolites was also changed. Basically, microbial fermentation in colon mainly includes saccharification fermentation and proteolytic fermentation (30). It is generally believed that saccharification fermentation is beneficial to the host, while proteolytic fermentation is presumed to be detrimental and may be involved in the metabolites (55). The primary product of carbohydrate fermentation (i.e., SCFA) has beneficial functions (56), and there was no difference between G1 and the control group. The metabolism of tryptophan bacteria leads to the production of a variety of indolic compounds (57). The concentration of 5-hydroxyindoleacetate was significantly increased. *In vitro* studies, indolic compounds had been shown to improve intestinal mucosal barrier function and reduce the expression of pro-inflammatory factor IL-8 (58, 59). The concentration of phenol in the G1 group was significantly decreased compared to controls. Some *in vitro* experiments had confirmed the damage of phenol to intestinal epithelial cells (60, 61). Phenol may transiently affect the lipid bilayer of the cell membrane, thereby destabilizing the microdomains containing tight junctions, suggesting that phenol is a potential driver of alterations in the gut barrier. Taken together, these results demonstrate that NMN increases the permeability of the intestinal mucosal by decreasing the concentration of phenol.

The increase of the FD4 leakage area and the expression of the tight junction protein show that long-term NMN treatment reduces intestinal permeability. Tight junctions limit flux of the paracellular pathway, which is generally more permeable than transcellular pathways. Therefore, tight junctions are the rate-limiting step of transepithelial transport and the primary determinant of mucosal permeability (62).

The increased expression of LC3 indicates that NMN promotes intestinal autophagy. Recent studies have shown that the integrity of the intestinal epithelial barrier is regulated by autophagy (63, 64). By establishing a starvation model, it was found that autophagy could induce the enhancement of tight junction, but only the paracellular flux of smallsized molecules decreased, and the transepithelial flux of large-sized paracellular probes was not affected. At the same time, it was found that the protein level of Claudin-2 was significantly down-regulated in the above experiments, and the localization was transferred from the membrane to the lysosome in the cytoplasm. It can be seen that autophagy enhances tight junctions by inducing the degradation of Claudin-2 under starvation (63). This suggests that NMN maintained mucosal barrier integrity by promoting autophagy to a certain extent.

Taken together, these results show that NMN maintains the integrity of the intestinal epithelium by strengthening tight connections and promoting mucus secretion. In conclusion, this study reveals that NMN can regulate the structure of the gut microbiota, increase the abundance of probiotics, and reduce the abundance of harmful bacteria. At the same time, it can also increase the concentration of bile acid-related metabolites in feces and decreases the concentration of phenols. NMN can also strengthen tight connections and promote mucus secretion. In this study, it exerted a protective effect on the intestinal mucosa, which had a positive effect on intestinal health. This study indicates a new direction for the use of NMN. However, the mechanism by which NMN regulates the gut microbiota has not yet been clarified, and further research is needed.

## Data Availability Statement

The datasets presented in this study can be found in online repositories. The names of the repository/repositories and accession number(s) can be found in the article/supplementary material.

## Ethics Statement

The animal study was reviewed and approved by Animal ethics committee of Jiangsu University.

## Author Contributions

PH and AJ designed and ran the experiment. YZ, XW, and WT performed the bioinformatics analysis. CR, ZZ, and XQ wrote and reviewed the manuscript. AG provided technical support. All authors contributed to the article and approved the submitted version.

## Conflict of Interest

The authors declare that the research was conducted in the absence of any commercial or financial relationships that could be construed as a potential conflict of interest.

## Publisher's Note

All claims expressed in this article are solely those of the authors and do not necessarily represent those of their affiliated organizations, or those of the publisher, the editors and the reviewers. Any product that may be evaluated in this article, or claim that may be made by its manufacturer, is not guaranteed or endorsed by the publisher.
